# Teaching Approaches for STEM Integration in Pre- and Primary School: a Systematic Qualitative Literature Review

**DOI:** 10.1007/s10763-023-10362-1

**Published:** 2023-03-16

**Authors:** Kevin Larkin, Thomas Lowrie

**Affiliations:** 1 School of Education and Professional Studies, Griffith University, 1 Parklands Drive, Southport, Gold Coast, Queensland 4222 Australia; 2 SERC, University of Canberra, Canberra, Australia

**Keywords:** Primary school STEM education, Models of STEM integration, Project-based learning, Inquiry-based learning, Problem-based learning, Play-based learning

## Abstract

In the last 5 years, there have been several literature reviews or meta-analyses investigating various aspects of STEM education; however, they have investigated a specific aspect of STEM, e.g. robotics, or digital games, or Early childhood, or Teacher perspectives. In addition, a broad-reaching review on STEM integration has not been conducted in the past 10 years. This article reports findings from a Systematic Qualitative Literature Review concerning STEM education for children aged 4–12 in formal education contexts. To provide context, the article initially presents descriptive findings (date and country of research, age of participants, research setting, and research methodologies used) in the 60 research articles that are included for analysis. The article then answers three research questions regarding the: (1) level of integration evident in the studies; (2) role of engineering in any such integration; and (3) teaching approaches used in the studies. Findings from this research suggest that there is still much work to be done to move from scenarios where STEM integration is claimed but is not evident in practice. To do so we encourage educators and researchers to (a) focus on authentic interdisciplinary approaches rather than the siloed approaches evident in the existing research; and (b) use a teaching approach such as problem-based or project-based learning that provide opportunities for authentic integration.

## Introduction

The purpose of this Systematic Qualitative Literature Review is to examine available research, in peer-reviewed journal articles (2000–present), regarding science, technology, engineering, or mathematics (STEM) integration in preschool and primary school contexts (children 4–12 years of age). Much of the current discussion regarding STEM revolves around an.economic rather than educational agenda. For example, in the Australian context, STEM education is part of a suite of measures that seek to make Australia a “science nation… in which science is woven, not only into our classrooms, but also into our boardrooms, our workplaces and our living rooms, as one of the building blocks of our prosperity” (Commonwealth of Australia, 2015, p. iii). Likewise, policy discourses in Europe and the USA increasingly position the importance of STEM in the educational context as the basis for future economic well-being (Marginson et al., 2013).

Whilst not ignoring the political and economic imperatives for STEM in current discourses, as educational researchers, we suggest it is of much greater importance to understand the educational implications of attempted STEM integration in educational contexts. We chose to focus on STEM education in pre-and elementary school (children 4–12 years of age) as (a) this is our area of research interest and expertise; and (b) research suggests that children’s attitudes to STEM disciplines is formed early in primary school (e.g., Larkin & Jorgensen, 2016) found that negative attitudes towards mathematics were already present in children 6–7 years old). A review into STEM integration is particularly timely, as although we are not the first to do so, a similar literature review regarding STEM integration is over ten years old (see Becker & Park, 2011). In addition, STEM integration is required if we are to avoid the siloed approaches to STEM education that are present in many of the articles discussed in this review.

To provide a background context for the reader, and to frame our three research questions (see “Method”), we briefly summarise some of the key literature in relation to the following three areas; STEM integration, engineering in STEM education, and approaches to teaching STEM.

### Theme One—STEM Integration

Although other methods for discussing STEM are present in the literature, for example, via teaching frameworks (see Greca Dufranc et al., 2020) or in terms of STEM content, pedagogy, or context (Cheng & So, 2020), the dominant approach used in the literature is one that frames STEM solely in terms of integration. For example, Dugger (2010) categorises STEM Integration into four categories: (a) four separate disciplines; (b) two of the four disciplines emphasised (e.g., SteM); (c) one discipline is integrated into the other three (e.g., E; STM), and (d) all four disciplines have equal emphasis and are approached in an interdisciplinary way. In a similar vein, Laksmiwati et al. (2020) present seven STEM perspectives including STEM as a very loose connection of the four separate disciplines; STEM as primarily Science, or Science and Mathematics, incorporating the other three or two disciplines respectively; through to full integration of the four disciplines. This full integration is seen by Nadelson and Seifert (2017) as a “seamless amalgamation of content and concepts” so that “knowledge and process of the specific STEM disciplines are considered simultaneously without regard for the discipline, but rather in the context of a problem, project or task” (p. 221).

Despite being implicit in the definitions of integration noted above, Vasquez et al. (2013) were explicit in expressing their view on the relative worth of the different forms of integration. They did so via the creation of a continuum of integration where less integrated forms of STEM are placed at the lower level of the continuum and fully integrated forms of STEM are placed at the highest level on the continuum. This continuum has frequently been used by researchers to assess the level of integration occurs when teaching STEM (see Anderson et al., 2019; Lowrie & Larkin, 2022). The continuum spans four forms of integration commencing with *Disciplinary* (concepts and skills are learned within each discipline); then *Multidisciplinary* (concepts and skills are still learned within each discipline but use a common theme); then *Interdisciplinary* (where concepts and skills from two or more disciplines are learned together); to the final *Transdisciplinary* form (where concepts and skills are learned across two or more disciplines but with a focus on real-world problems). Given that the Vasquez et al. (2013) continuum is used in many of the articles we reviewed, we decided to base our initial analysis of integration in the articles in this study using this continuum.

### Theme Two—STEM or Just STM

Although the acronym STEM has been evident in educational contexts for the last two decades (see Lowrie & Larkin, 2022), its enactment in schools has not been as pronounced, and this is particularly true in the case of the E in STEM. Research on engineering education, as part of the broader STEM education agenda, is limited and perhaps reflects, at least in school contexts, the rather problematic nature of engineering as one of the STEM disciplines. This problematic nature can be accounted for from at least three perspectives; namely curriculum, pedagogy, and gender.

As a curriculum example, in the Australian educational context, while there are clearly defined science, mathematics and technology curriculums, there is no specified engineering curriculum. Instead, the Australian Curriculum, Assessment and Reporting Agency (ACARA), argues that “engineering is addressed across the curriculum through Science, Technologies and Mathematics and in a dedicated content description focusing on engineering principles and systems at each band in Design and Technologies” (Australian Curriculum Assessment and Reporting Authority [ACARA], 2017). Whilst this may appear to be a good outcome in terms of integrating engineering into other STEM disciplines, from our experience, when a curriculum area is intended to be integrated across a range of areas it often means, in practice, it is not integrated into any of them. As Lowrie and Larkin (2020) illustrate, this approach is problematic as the individual disciplines are often written independently of each other, with little attempt at developing authentic connections between them. Given this observation, the lack of a dedicated engineering curriculum within a curriculum suite, places engineering in primary schools in a precarious position.

In terms of pedagogical issues related to the teaching of engineering, Dubosarsky et al. (2018) indicate that “one of the reasons for the lack of STEM and engineering instruction is educators’ low self-efficacy regarding the teaching of STEM, due in part to a lack of preparation and shortage of early childhood STEM and engineering curricula” (p. 252). This lack of confidence in teaching engineering occurs, in large part, because engineering is as new for most early childhood educators as it is for children (Cunningham et al., 2018).

An urgent requirement for the better teaching of engineering in the early and primary years is, therefore, the professional development of educators. Lippard et al. (2018) advance the argument that professional development in mathematics and English has encouraged early years educators to recognise early literacy and maths skills and to value these “pre” skills. Likewise, they claim “teachers will, similarly, need training and support to recognize and appreciate pre-engineering skills” (p. 32). English (2018) also identifies concerns and calls for professional development that can “assist teachers in better understanding the nature and role of engineering learning, together with effective planning and the enactment of integrated STEM lessons” (p. 282). In a somewhat novel solution, Estapa and Tank (2017) promote a triadic method of professional development with the triads consisting of a classroom educator, a pre-service educator, and an engineering fellow, where the focus is on enhancing knowledge of STEM concepts using an engineering design approach. These authors argue that any real changes in practice call for “sustained, coherent, collaborative, reflective teacher programs” (p. 2).

Finally, in terms of gender, as a logical consequence of the limited research overall, there is only a limited body of research on the role that gender plays in engineering education in the early and primary years. Sullivan and Bers (2016) indicate that, even though the gender disparity has decreased over the past decade or so, this disparity remains at its largest in terms of engineering opportunities. Research by Pattison et al. (2016) highlights the “critical need to understand how engineering interests develop and can be supported before children enter school; and the effectiveness of supporting, through early childhood interventions, long-term engineering-related interest development” (p. 1). In some brighter news, Metz (1997), and Greca Dufranc et al. (2020), found that robotics and computer programming, particularly in the early years, can provide girls with positive experiences of engineering before negative gender stereotypes begin to set in during the upper primary school years.

Given the rather glum picture we have painted in relation to the place of engineering in STEM, in terms of curriculum, pedagogy, and gender, we were interested to test our research question that E would be underrepresented in the STEM articles that form the dataset for this article.

### Theme Three—STEM Teaching Approaches

We have identified, in previous work in this domain, (see Larkin & Lowrie, 2022; Lowrie, et al., 2017) that three teaching approaches are typically associated with STEM education: Inquiry-based, Project-based, and Problem-based, learning. In examining the articles in this review, we discovered that in some of the articles, the related term, Design-based learning, was used (see Bagiati & Evangelou, 2015; Sariçam & Yildirim, 2021). In instances where this occurred, we looked to see whether the projects related to real-world issues generated by the children or whether they were issues proposed by the teacher, and then categorised them as Problem-based or Project-based respectively. Regardless of the terminology used, this group of learning approaches has been identified as being beneficial for students in terms of a) cognitive aspects, e.g. improved connectivity between discipline areas (Estapa & Tank, 2017) and increases in higher-order thinking skills and creativity (Fan & Yu, 2017); and b) affective aspects— (for example, positive changes in perceptions of STEM-related careers and disciplines (Knezek et al., 2013). We briefly outline the features of each of the three approaches below.

#### Inquiry-based learning

According to Bybee (2010), descriptions vary in terms of what Inquiry-based learning means, and these differences are often represented using a continuum from educator-directed to child-centred approaches (Anderson et al., 2019; Calder et al., 2020). At the educator-directed end of the continuum, there is minimal inquiry (e.g., where an educator provides explicit instructions regarding how children are to carry out an experiment or investigation). At the child-centred end is an open-ended inquiry where children initiate both their own questions and their own processes to answer those questions. Albion (2015) notes that, whilst sharing many features of Project-based and Problem-based learning, a distinguishing feature of Inquiry-based learning is that it follows a cyclical scientific method that is often expressed in terms of the 5Es*—Engage, Explore, Explain, Elaborate*, and *Evaluate* (Bybee, 2010). Thus, Inquiry-based learning can take place over a shorter period, with greater scaffolding by educators, than is likely in either of the other two learning approaches.

#### Project-Based Learning

Lowrie et al. (2017) indicate that Project-based learning involves children investigating a particular problem, question, or challenge for a sustained period. Several key features characterise this approach and these include educators:

- Identifying authentic problems that are likely to be of interest to children (Estapa & Tank, 2017)
- Assisting children to establish connections between these problems and their real-life experiences
- Supporting children to solve these problems using the concepts they have been learning (Dierdorp et al., 2014)
- Encouraging the creation of a meaningful product (Albion, 2015)

#### Problem-Based Learning

Problem-based learning is similar in many respects to Project-based learning as both involve children working to solve open-ended problems. The distinguishing feature of Problem-based approaches are that they relate to the children’s real-life experiences, are posed by the children, and aim to challenging them to think differently in finding solutions (English & Mousoulides, 2015). To facilitate an understanding by children, regarding how STEM-based knowledge and skills work outside the classroom, a necessary component of Problem-based approaches is that they offer multiple solutions and pathways to success (English & Mousoulides, 2015) and normally occur over a period of at least several weeks (Albion, 2015).

## Research Questions

Based on our work in STEM in the before school, early years, and primary years of schooling (see Larkin & Lowrie, 2022; Lowrie et al., 2017, 2019) we approached this review with three research questions in mind:

- *RQ 1#. Do most articles included in the dataset involve only the lower levels of integration (i.e. Disciplinary or Multidisciplinary as opposed to Interdisciplinary or Transdisciplinary)?*
- *RQ 2. Is engineering the least integrated of the four individual STEM disciplines?*
- *RQ 3. Are most teaching approaches either Inquiry-based, Problem-based, or Project-based?*

We will return to these research questions later in the article. Initially, we explain the methodology we followed to find relevant research according to our selection criteria, and then we explore some of the descriptive findings from the Systematic Qualitative Literature Review.

## Method

### Search Strategy Used

In finding STEM published research, we followed the established Preferred Reporting Items for Systematic Reviews and Meta-Analysis (PRISMA) search protocols (Page et al., 2021). For consistency with previous Systematic Qualitative Literature Reviews, we used databases that have been used in previous research by Becker and Park (2016), Sullivan and Heffernan (2016), and Margot and Kettler (2019). The databases searched were ERIC; Informit + ; Proquest; Sage Journals; and Taylor and Francis Online. The following two searches were conducted in each of the five databases:

- ti((child* OR preschool OR Kindergarten OR early childhood OR School)) AND ti(STEM) AND all(STEM) stype.exact(“Scholarly Journals”) AND PEER(yes) since 2000
- (ti((child* OR preschool OR Kindergarten OR early childhood OR School)) AND ti(Science AND Technology AND Engineering AND Mathematics) AND stype.exact(“Scholarly Journals”) AND PEER(yes)) AND stype.exact(“Scholarly Journals”) AND PEER(yes).

**Fig. 1:**
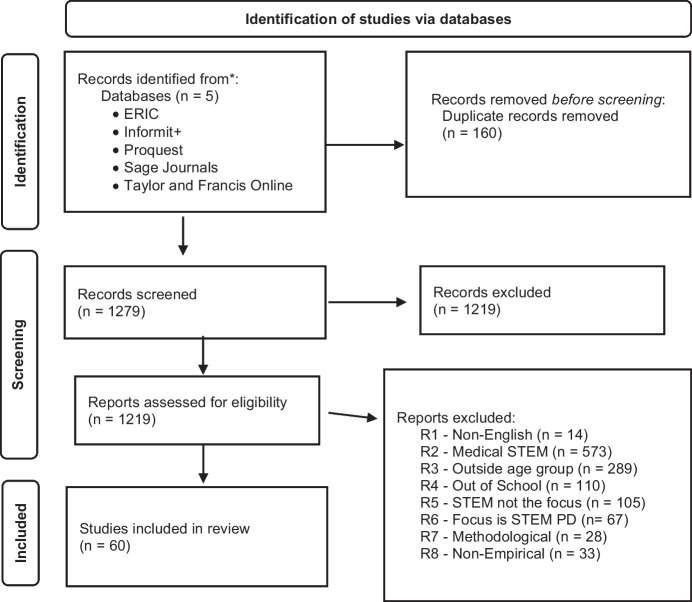
Identification of studies via databases (adapted from PRISMA*—*see Page et al., 2021)

The search was limited the research to peer-reviewed journals articles between 2000 and 2022. The year 2000 was chosen as it was then that STEM became a topic of focus in education. To maximise replicability of our search strategy, we choose to only include peer-reviewed research published in journals. Following this process, we found 1439 articles and, after 160 duplicates were removed, we were left with 1279 articles for further screening (see Fig. 1).

### Details regarding screening process

As recommended in the PRISMA Statement (Page et al., 2021), the screening process followed several steps. Initially, we examined the titles, abstracts, and keywords to determine whether the research met our criteria for inclusion (see Table 1) and to exclude those that did not.

**Table 1:** Criteria for inclusion and exclusion

*Inclusion criteria*	*Exclusion criteria*
Published in English	Non-English language publications
STEM in educational contexts	STEM in medical contexts
Focus is on preschool or primary school children	Age of participants outside of this range or age of participants not established
Focus of research is “in-school” STEM	Out of school research (e.g., Museum visits, Summer Camps)
Focus is on STEM education	STEM is a vehicle for other goals (e.g., Entrepreneurship or STEM employment)
Focus is primarily on school aged children’s STEM learning	Focus is primarily Teacher PD or Preservice Teacher Education Focus is related to methodological concerns (e.g., validity testing, meta-analyses
Empirical research	Non-empirical research

Following this initial scan, we were left with 109 articles. The next step of the process involved reading these 109 articles in full. This was necessary to accurately answer our three research questions, and to clarify the suitability of articles where our inclusion criteria were not clear from the title, abstract or keywords. This process resulted in a further 49 articles being excluded, which left us with 60 articles included for descriptive analysis (see “Appendix” for full bibliographic details).

Three observations and clarifications are important at this juncture.

1. In Fig. 1, we have included the list of reasons for non-inclusion in the study. The list indicates the order that the exclusion criteria were applied and, therefore, only demonstrates one potential pathway in determining the suitability of the articles. If the pathway was modified, the number of articles deleted at each step would change; however, the final number of 60 articles that meet all inclusion criteria (i.e. none of the exclusion criteria) would remain the same. For example, some of the articles excluded on the “Outside Age Group” criteria concerned “Out of School” STEM and thus, if the steps were reversed, there would be a larger *n* for “Out of School” STEM.
2. We stress that we are not suggesting, by their exclusion, that “Out of School” STEM activities are not beneficial; indeed, in Larkin and Lowrie (2022) we argue for their important role in supporting STEM learning. Likewise, as we have argued elsewhere, professional development for teachers is critical (see Larkin & Lowrie, 2022; Resnick et al., 2022). However, as the focus of our three questions include levels of integration and types of pedagogical approaches, it was necessary to limit our focus to children’s in-school STEM learning.
3. Some of the final 60 papers also include aspects of the exclusion criteria. For example, both Cotabish et al. (2013) and Dejarnette (2018) discussed professional development as one element of their research; however, each of these research papers had as their primary focus, elements that met inclusion criteria. In instances like these, these articles were included in the review.

Although the large bulk of articles initially found in the database search were excluded, the number of articles left to address our three questions is, when compared with other STEM related Systematic Qualitative Literature Reviews (see Table 2), quite large.

**Table 2:** Related STEM Education Systematic Qualitative Literature Reviews

*Author and year*	*STEM topic of interest*	*No. of articles included in review*
Sullivan and Heffernan (2016)	Robotic Construction Kits as Computational Manipulatives for Learning in the STEM Disciplines	41
Wahyuningsih et al. (2020)	STEAM Learning in Early Childhood Education	40
Wang et al. (2022)	Effects of digital game-based STEM education on students’ learning achievement	33
Becker and Park (2011)	Effects of integrative approaches among STEM subjects on students’ learning	28
Margot and Kettler (2019)	Teachers’ perception of STEM Integration and Education	25
Wan et al. (2020)	STEM Education in Early Childhood	24
Çetin and Demircan (2020)	Empowering technology and engineering for STEM education through programming robots	23
Ng et al. (2022)	Integrating and navigating STEAM in early childhood education	17
Tselegkaridis and Sapounidis (2022)	A Systematic Literature Review on STEM Research in Early Childhood	16
Yücelyigit and Toker (2021)	A meta-analysis on STEM studies in early childhood education	5

#### Compilation of the Data

**Fig. 2:**
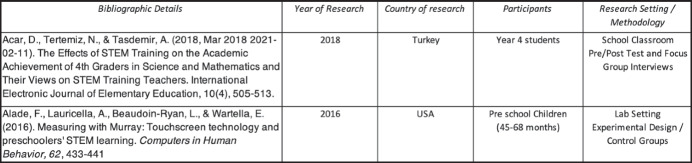
Sample of descriptive information

**Fig. 3:**
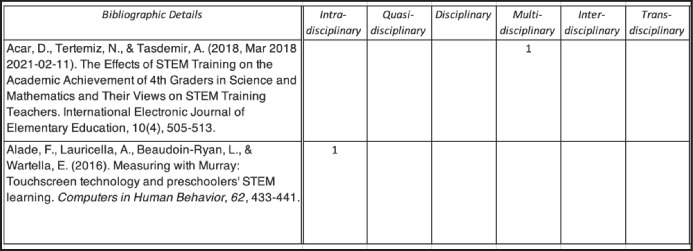
Sample of levels of integration

**Fig. 4:**
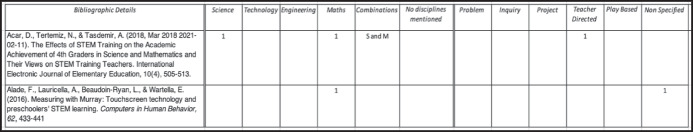
Sample of discipline combinations and learning approaches

Once the final 60 articles were determined, we then began a comprehensive data collation process and used an Excel spreadsheet to assist in the compilation and analysis of the data. To situate the research articles in context, we identified several types of descriptive statistics (see Fig. 2): namely, year of publication, country where research was conducted, the participants in the study, and the research setting. We also coded data according to our three research questions (see Figs. 3 and 4). As articles often included several data collection techniques (e.g., interviews, surveys, classroom observations), some articles were counted twice, using two difference codes (e.g., as using interviews and as using pre and post measures). One further point of clarification is required. Rather than determining ourselves whether a STEM activity was, for example, engineering or technology (given the close relationship between the two), we relied on the terminology used by the original researchers in their depictions of whether an activity was engineering or technology. The spreadsheet containing the full coding information is available at this link (https://www.dropbox.com/s/nwder8jpc4j9ecu/Raw%20Data%20for%20online%20file.xlsx?dl=0).

## Descriptive Analysis

**Fig. 5:**
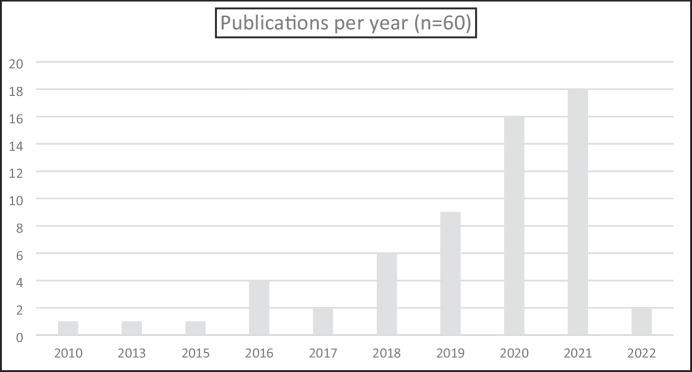
Publications per year since 2000

To provide an overall view of the research, we now discuss the descriptive statistics we collected that provide a holistic context for the remainder of the article. As we indicated earlier in our search criteria, as STEM as an educational issue largely coincided with the start of the new millennium, we searched for articles in the period of 2000–2022. It was a little surprising to find that the first peer-reviewed article relating to either preschool, early years or primary STEM was only published in 2010, with the bulk of the articles appearing in the last four years (see Fig. 5).

The large increase in the number of publications towards the end of the last decade possibly reflects various national government initiatives to improve national economic and social mobility outcomes via a STEM agenda. For example, in the Australian economic context, “an improvement of 1% in STEM related roles could add $57.4 billion to GDP” (Australian Academy of Science, 2016, p. 4). Maass et al. (2019) note that in the European context, “it is also increasingly recognised that Science, Technology, Engineering, and Mathematics (STEM) education is an essential foundation for responsible citizenship and the ethical custodianship of our planet” (p. 870). The larger number of the articles towards the end of the period under investigation could also reflect the findings of Li et al. (2020) who report that funded STEM research in the USA was unevenly distributed. As the trend for average funding of STEM research reached a peak in 2014, it could be the case that this accounts for a peak in publications in the years following the conclusion of the projects.

**Fig. 6:**
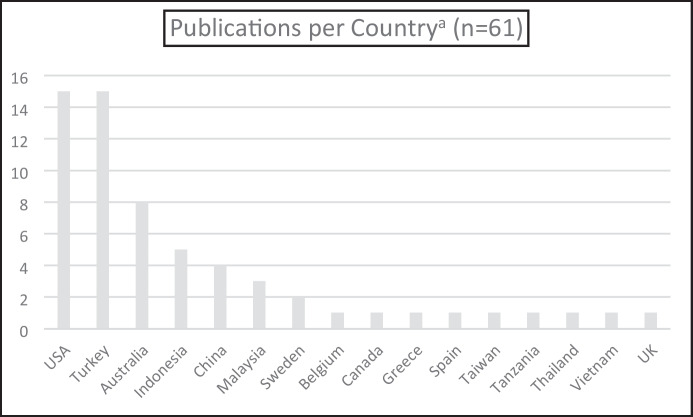
Location of Research. ^a^Publications total 61 as one study was conducted in two countries

We also coded the articles to determine the countries in which the research took place (see Fig. 6). All but one of the articles involved research in a single country, with the exception being an article by Greca Dufranc et al. (2020) involving a case study approach using classes in Spain and Sweden.

As STEM originated in the USA, we were not surprised that many studies took place there; however, contrary to the findings of Margot and Kettler (2019) regarding teacher perspectives of STEM integration, where 20 of the 25 studies in their review were from the USA, the studies in our review were more evenly distributed (see Fig. 6). As Australian based researchers, we were aware of various research projects in our educational “backyard”; however, what was surprising to us were the large number of studies conducted in Turkey. We surmise that the 15 articles reporting research in Turkey, each published from 2018 onwards (with 13/15 published in last two years), may have been a consequence of a report regarding STEM Education in Turkey (Bücük, 2016) where the Minister of Education called for the development of a dynamic education system that would “raise a generation who will invent scientifically in the future” (n.p.).

One of the inclusion criteria was STEM research involving primary school aged children (4–12 years old). As seen in Table 3, the articles reported a mixture of child only (26/60), teacher only (12/60), and both children and teachers, as participants (22/60). Recall that, to be included, the articles with only teachers as participants needed to focus on curriculum or pedagogy and not only professional development. Although none of our research questions delineate between stages of primary school, for transparency (see Table 4), we report the split of studies according to preschool children only (18/48), primary school children only (26/48), or both groups (4/48) Most of the primary school research occurred in the first four years of schooling (i.e. 4–8-year-olds).

**Table 3:** Study participants (*n* = 60)

Children only	26
Both children and teachers	22
Teachers only	12

**Table 4:** Studies including children (*n* = 48)

Preschool children only	18
Primary school children only	26
Both preschool and primary school children	4

As seen in Table 5, there was a broad range of research methodologies employed in the articles, ranging from more quantitative empirical studies (pre- and post-tests and/or experimental/control groups), mixed methods (surveys and document analysis), through to the more qualitative approaches of (interviews, focus groups, classroom observation and work samples). Sample sizes of the studies ranged from 12 to 1201 participants, with four large studies each with more than 800 participants, and nine small studies each with less than 20 participants. In terms of average number of participants, the mean was 139, the median 44, and the mode 40.

**Table 5:** Approaches to data collection (*n* = 115)

Pre and post measures	15
Experimental/control groups	13
Surveys	17
Interviews	29
Focus groups	3
Work samples	10
Classroom observation	21
Document analysis	7

Number is greater than 60 as many studies used more than one approach to data collection

## The Three Research Questions

We now examine the data collected from the articles to answer each of the three research questions indicated earlier in the article.

### RQ 1#. Do most articles included in the dataset involve only the lower levels of integration (i.e.Disciplinary or Multidisciplinary as opposed to Interdisciplinary or Transdisciplinary)?

**Fig. 7:**
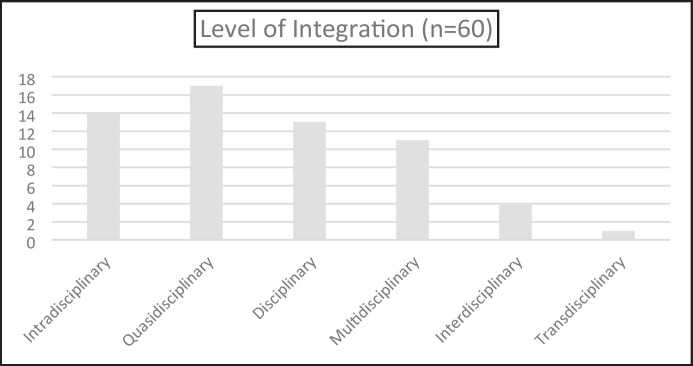
Level of Integration of STEM disciplines

Given the widespread use of the Vasquez et al. (2013) model for categorising STEM integration (see Anderson et al., 2019; Larkin & Lowrie, 2022), we commenced an examination of the types of STEM integration using this model as a coding device. However, it became apparent early in the coding process that there were claims of integration in the research articles that did not, in our view, correspond to any of the four levels of integration proposed by (Vasquez et al., 2013) (see Fig. 7), and thus, new categories were required.

Firstly, we found articles that, although they included STEM or STEM integration in the title, did not demonstrate any (or at least only very incidental) integration between disciplines. These examples of research “within” a sole STEM discipline have been coded for this analysis using the term *Intradisciplinary*. Examples of *Intradisciplinary* research include the use of a tool (e.g., iPads (Aladé et al., 2016)), or a STEM discipline methodology (e.g., the 5Es in science (Ültay et al., 2020)), within the domain of one individual discipline. In instances such as this, the term STEM in the article title might more accurately be replaced with a specific discipline term such as science, engineering, etc.

The second category not included in Vasquez et al. (2013) is similar to the Intradisciplinary category in that a specific STEM discipline methodology is used; however, in this instance, the methodology is used to (a) teach either content from another specified STEM discipline (e.g. an Engineering Design approach is used to teach mathematics (Firdaus et al., 2020; Gold et al., 2021)); or (b) the methodology is used to teach STEM without identifying any disciplines (e.g. an Engineering Design approach is used to teach STEM (Malcok & Ceylan, 2021)). This category also includes the use of a non-STEM specific instructional approaches to teach STEM (e.g., play-based learning (Stephenson et al., 2021) or blended learning (Seage & Türegün, 2020)). For cases such as these, the term *Quasidisciplinary* is used. We have two observations to make regarding these findings, which touch on issues related to either end of the Vasquez et al. (2013) continuum. The first relates to the lack of studies at the more integrated end of the continuum and the second relates to the need to create two new categories at the lower end of the continuum to accurately reflect the level of, or lack of, integration.

### Lack of Interdisciplinary and Transdisciplinary Integration

Of the 60 studies analysed in this study, only 5/60 (or ≈ 8%) involved the higher levels of integration i.e. *Interdisciplinary* or *Transdisciplinary*. Koul et al. (2018) provide evidence of transdisciplinary integration in their project, which involved children completing a selection of design-based problems (e.g., investigating bike wheel friction, an oil spill, or the workings of solar panels). What makes this work transdisciplinary is that, in each of the problems, there was an emphasis on concepts from each of the four disciplines and how these need to work together to generate potential solutions to the problems. The observation that most projects demonstrated low levels of interdisciplinary integration is perhaps unsurprising given the lack of content and pedagogical knowledge regarding STEM for primary and early childhood educators (Fleer, 2009); and curriculum design issues where individual STEM disciplines have their own curriculums (Engineering being an exception) with little thought as to how these disciplines might be taught in integrated ways (Lowrie & Larkin, 2020). Although we had anticipated difficulties with the higher order integration of STEM, it is a little surprising, given the greater flexibility with timetabling (Honey et al., 2014), and the fact that primary school teachers normally teach several subjects (Shernoff et al., 2017), that we did not see more studies involving evidence of the higher levels of integration in this analysis.

### Intradisciplinary and Quasidisciplinary Integration

An initial finding was that less than 50% of the studies (29/60) reflected any of the four levels of integration as proposed by Vasquez et al. (2013). To accurately reflect the level of integration in 31 of the studies, which did not even reach the lowest integration level (*Disciplinary*)—where students learn concepts and skills separately in each discipline—it was necessary for us to create two even less integrative categories—*Intradisciplinary* (effectively STEM is claimed, but only one discipline is involved) and *Quasidisciplinary* (STEM is again claimed, but it is just the use of one discipline methodology—or a non-STEM methodology—to teach a STEM discipline). Possible causes for these findings relate to the issues raised above regarding the difficulties of integration. However, they may also reflect the fact that the STEM agenda has become a powerful source of research and school funding in the last decade or so, and this funding is only attracted by STEM badged research or teaching activities. Thus, the incentive is there to claim STEM integration, even though this integration is only minimal, with the primary thrust of the research appearing to involve only one discipline. This incentive is also evident in the plethora of STEM siblings—STEAM, STEMM, STEMR, STEM C + (see Lowrie et al., 2017), where hitching a ride on the STEM train can mean additional financial support for research and teaching endeavours.

### RQ 2. Is engineering the least integrated of the four individual STEM disciplines?

**Fig. 8:**
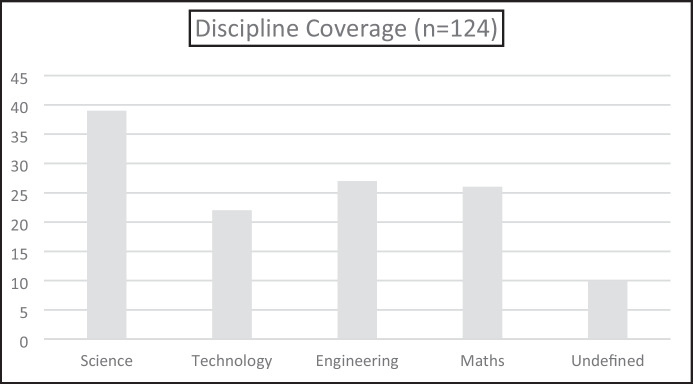
Integration of STEM by discipline

Given our experiences in STEM education in Australia, and our knowledge of research in the international STEM context, we anticipated that engineering would be the least integrated STEM discipline. However, as depicted in (Fig. 8), this was not the case, with engineering and mathematics almost equal as the second most integrated disciplines after science with technology being the least integrated discipline.

**Fig. 9:**
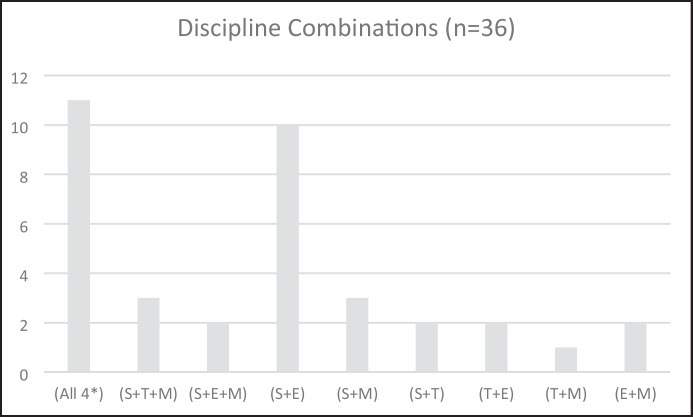
Combinations of STEM disciplines. *Indicates that each of the four disciplines was specifically mentioned and excludes articles that indicated STEM integration but did not name any specific discipline

Figure 8 is a raw count of the number of times the individual disciplines were named in the articles reviewed. We were also interested to see the pattern of combinations of the disciplines as, given that some of the articles were not really about integration at all, this might give us more of a sense of which disciplines are more easily, or at least more regularly, integrated than others. These combinations are provided in (Fig. 9). For the purposes of this discussion, we are not proposing any evaluation of the relative worth of the disciplines in the order that they are presented and for convenience, we have listed the combinations in the order that the letters appear in the word STEM. For example, a study about engineering and science and a study about science and engineering are both included in the S + E category, with no implication that the discipline listed first was the primary one. Secondly, to be coded as “(all 4*)”, the articles needed to specifically mention the four disciplines. This was used as a quality control measure, as ten of the 60 articles reviewed just referred to STEM throughout, without specific mention of the different disciplines or how they were integrated. This meant it was impossible for use to assess discipline integration in the articles and thus they were coded as “undefined”.

In terms of the various combinations of disciplines, excluding the instances where all four disciplines were combined, the results show that science was included in 20 combinations, engineering in 16 combinations, mathematics in 11 combinations, and technology in 8 combinations. The science and engineering combination is interesting as they were paired to a much greater degree than any other pairing. The combinations of disciplines found in our review can be compared with the meta-analysis of 28 articles by Becker and Park (2011), who also investigated integrative approaches to STEM education present in the literature, albeit with a broader age range (students were from elementary through to college age). These authors report that ten of their studies involved integration of (S + M); three combinations had five studies each (S + T + E; S + T + M; and S + T); and three further combinations had one study each (S + T + E + M; E + M; and S + E).

The main differences between the results of Becker and Park (2011) and our results are in the integration pairs of (S + M) with 10/28 and 3/36 respectively; (S + T + E + M) with 1/28 and 11/36 respectively) and (S + E) with 1/28 and 10/36 respectively. There are several possible explanations for the differences. Firstly, the meta-analysis by Becker and Park (2011) investigated the impact of STEM on student learning and thus science and mathematics may have figured more prominently, as these are established curriculum subjects, with historical standardised tests that can be used to measure learning. In addition, the stronger emphasis on engineering in our review might relate to the observation that engineering was under researched in early STEM publications. Given it is more than ten years since the Becker and Park (2011) study, the rebalancing of emphasis on engineering might account for this difference. In our analysis, 21 of the 27 studies that included engineering were published in the last 3.5 years. What is curious to us is that only one study in the research of Becker and Park (2011) involved all four disciplines. This could again be related to the earlier observation that science and mathematics have a long history as school curriculum subjects, with technology (at least in its digital aspects) a latecomer in a curriculum sense (see Lowrie & Larkin, 2020) and engineering already established as being the “difficult child” in the STEM family.

### RQ 3. Are most teaching approaches either Inquiry-based, Problem-based, or Project-based

Initially, a point of clarification is required regarding how we made our classifications. In some of the articles the author(s) indicated that they used one of our three approaches; however, if we determined, according to our classification system, that the articles portrayed a different approach, we categorised the article according to our evaluation and not according to the author(s) determination. By way of example, Ergün and Külekci (2019) indicate in their article that they used a Problem-based approach; however, in our view, it only minimally involved this approach and was primarily a series of science lessons regarding Friction and Force. Consequently, we categorised this article as using a teacher directed approach.

**Fig. 10:**
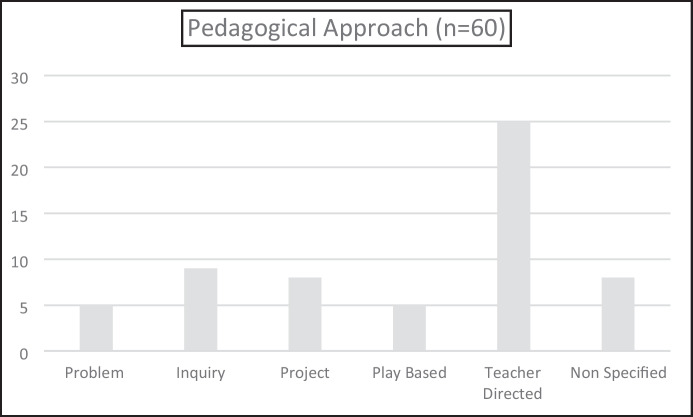
Pedagogical approach used in research

Our initial coding, using the three types of approaches (Problem-based, Inquiry-based, and Project-based), proved inadequate for the task of categorising learning approaches in the studies reported upon here. If we coded the articles using only the original three approaches, 38 out of the 60 studies would have been coded as “Other”. Therefore, we added a further two codes; namely, play-based, and teacher directed, approaches (see Fig. 10). As we have already defined Problem-based, Inquiry-based, and Project-based approaches, we will now briefly define Play-based, and Teacher-directed, approaches. By Play-based we mean instances where children are involved in learning about STEM as they engage in play. Although a difficult concept to define, our emphasis in this classification is on STEM learning through play and can be seen, for example, in Stephenson et al. (2021) and Tippett and Milford (2017). By Teacher-directed approaches we mean scenarios where teachers plan for STEM learning or STEM integration but do not provide opportunities for children to have any agency in this learning or integration. In our classification this is seen, for example, in Yavuz and Yildiz Duban (2021) and Türk and Akcanca (2021).

As was the case with our earlier discussion regarding the reasons for low levels of integration, we suggest that the large number of teacher directed approaches evident in the research (25/60 or 42%) again relate to issues concerning curriculum, where discipline content are developed separately with minimal attempt at integration; and concerns regarding the content and pedagogical knowledge of educators in relation to some or all of the STEM disciplines. These issues then impact on how researchers can investigate STEM in school contexts. The distribution of the three types of STEM approaches we had predicted was relatively equal, with Problem-based learning being the least common of the three approaches. This likely reflects that Problem-based learning is the more difficult of the three for teachers to implement as this type of learning requires teachers to “hand over” a significant amount of learning control to the children, and then having the pedagogical and content knowledge confidence to be able to guide the children in their learning rather than the more teacher directed aspects of Inquiry-based and to a lesser extent Project-based learning (see Anderson et al, 2019).

The prevalence of play-based learning is accounted for in two ways; firstly, a number of the articles reported on research with pre-school children and play-based learning is a dominant pedagogy in this age group; secondly, two of the articles involved the research of Fleer concerning Conceptual Play Worlds, which, by definition incorporate play-based methodologies. As we have reported elsewhere (Larkin & Lowrie, 2022) we agree with Fleer that play-based learning, when accompanied by an appropriate level of educator intentionality, is a robust way to support children’s STEM learning beyond preschool and into the early years of formal schooling.

## Limitations

Whilst the search criteria resulted in an initially large set of research articles, the criteria of only selecting peer reviewed journal articles means that we might have missed important research published in book chapters or government reports, or relevant work presented at conferences. We also only reviewed STEM research that directly focussed on children in formal contexts, so again we may have overlooked important insights from work that focussed on STEM in out of school contexts, the ongoing professional development of teachers, or the training of pre-service teachers.

A second limitation relates to our specific focus on the actual integration of STEM, the role of engineering within any integration, and the various teaching approaches used to deliver such integration. Thus, many of the articles we reviewed provided important insights regarding STEM (e.g. supporting creativity (see Sariçam & Yildirim, 2021) or STEM and gender (see Sullivan & Bers, 2016))—that were outside the scope of our three research questions. Thus, some of the articles that we have been somewhat critical of, make contributions to the STEM research field, but do so outside of our focus.

## Conclusion

This Systematic Qualitative Literature Review was the first in over ten years to investigate peer reviewed research regarding STEM integration, and the first that focused solely on the primary school years of schooling (4–12 years). Our initial search of a large body of articles found over 1200 potentially relevant articles; however, only 60 articles met our STEM integration inclusion criteria. Given the amount of funding for STEM in the last two decades, we had anticipated that more research into STEM integration may have occurred with primary age students. We particularly encourage more research into the integration of STEM in the middle and upper primary school age group, as research with this subset of primary-aged students was underrepresented in the 60 studies evaluated in this article. A second recommendation, based on our analysis, would be for researchers to utilise existing STEM performance measures (see Lin et al., 2021; Malcok & Ceylan, 2021) when investigating STEM integration. In much of the research, this is not the case. In Acar et al.(2018) and He et al. (2021), for example, existing measures of science, mathematics, or technology achievement are used as a means of measuring STEM achievement. In our view, this is approach counterproductive, as measures of the independent disciplines, are likely to reinforce the siloed positions of the four disciplines and thus hinder more authentic STEM integration.

In examining the articles, we also viewed them through the lens of several research questions that we generated based on our experiences as STEM researchers and educators. These research questions focussed our attention on whether: claims of STEM integration, in the titles and abstracts, were supported by evidence in the body of the articles; the articles were STEM or merely STM; and there were established STEM teaching methodologies evident in the research studies. In terms of STEM or STM, we found that there has been an increased emphasis on research regarding the E in STEM, with 27 studies including engineering. In addition, many of these studies used what was described as an engineering design process. Using this design approach could bear fruit in classrooms, as it is one that can easily support integration with other disciplines (see Bagiati & Evangelou, 2015), as well as provide opportunities for greater student agency in their own learning (see Dubosarsky et al., 2018).

Whilst the finding regarding an increased role for engineering is a positive for STEM education, what we discovered in relation to the remaining two research questions suggests that much work remains to be done if the rhetoric of STEM integration is to be matched in practice. In our view, two main agenda items remain to be addressed. Firstly, how can teachers, based on more highly integrated research into STEM, be supported in providing children with STEM experiences that are either *Interdisciplinary* or *Transdisciplinary* (as STEM is envisaged and promoted) rather than what is currently occurring where STEM is, at best, taught in only *Disciplinary* or minimally *Multidisciplinary* ways. Secondly, how can teachers be supported in moving beyond Teacher-directed types of STEM learning (that likely hamper attempts at authentic integration) towards Inquiry-based, Problem-based, Project-based, or Play-based approaches, which are better placed to support the integrative intent of STEM.

## Appendix. List of articles used in this SQLR

Acar, D., Tertemiz, N., & Tasdemir, A. (2018). The effects of STEM training on the academic achievement of 4th graders in science and mathematics and their views on STEM training teachers. *International Electronic Journal of Elementary Education, 10*(4), 505–513. https://www.iejee.com/index.php/IEJEE/article/view/465

Alade, F., Lauricella, A., Beaudoin-Ryan, L., & Wartella, E. (2016). Measuring with Murray: Touchscreen technology and preschoolers’ STEM learning. *Computers in Human Behavior, 62*, 433–441. 10.1016/j.chb.2016.03.080

Amran, M., Bakar, K., Surat, S., Mahmud, S., & Shafie, A. (2021). Assessing preschool teachers’ challenges and needs for creativity in STEM education. *Asian Journal of University Education, 17*(3), 99–108. 10.24191/ajue.v17i3.14517

Anderson, J., Wilson, K., Tully, D., & Way, J. (2019). “Can We Build the Wind Powered Car Again?” Students’ and teachers’ responses to a new integrated STEM curriculum. *Journal of Research in STEM Education, 5*(1), 20–39. 10.51355/jstem.2019.61

Awang, Z., Yakob, N., Hamzah, A., & Talling, M. (2020). Exploring STEAM teaching in preschool using Fred Rogers approach. *International Journal of Evaluation and Research in Education, 9*(4), 1071–1078. 10.11591/ijere.v9i4.20674

Bagiati, A., Yoon, S., Evangelou, D., & Ngambeki, I. (2010). Engineering curricula in early education: Describing the landscape of open resources. *European Early Childhood Education Research Journal, 23*(1), 112–128. 10.1080/1350293X.2014.991099

Blackley, S., & Howell, J. (2019). The next chapter in the STEM education narrative: Using robotics to support programming and coding. *Australian Journal of Teacher Education (Online), 44*(4), 51–64. 10.14221/ajte.2018v44n4.4

Campbell, C., Speldewinde, C., Howitt, C., & MacDonald, A. (2018). STEM practice in the early years. *Creative Education, 99*, 11–25. 10.4236/ce.2018.91002

Çetin, A. (2020). Examining project-based STEM training in a primary school. *International Online Journal of Education and Teaching, 7*(3), 811–825. https://iojet.org/index.php/IOJET/article/view/761

Chaldi, D., & Mantzanidou, G. (2021). Educational robotics and STEAM in early childhood education. *Advances in Mobile Learning Educational Research, 1*(2), 72–81. 10.25082/AMLER.2021.02.003

Ching, Y., Yang, D., Wang, S., Baek, Y., Swanson, S., & Chitoori, B. (2019). Elementary school student development of STEM attitudes and perceived learning in a STEM integrated robotics curriculum. *TechTrends, 63*(5), 590–601. 10.1007/s11528-019-00388-0

Cotabish, A., Dailey, D., Robinson, A., & Hughes, G. (2013). The effects of a STEM intervention on elementary students’ science knowledge and skills. *School Science & Mathematics, 113*(5), 215–226. 10.1111/ssm.12023

Counsell, S., & Geiken, R. (2019). Improving STEM teaching practices with R&P: increasing the full range of young children’s STEM outcomes. *Journal of Early Childhood Teacher Education, 40*(4), 352–381. 10.1080/10901027.2019.1603173

Dedetürk, A., Kırmızıgül, A. S., & Kaya, H. (2021). The effects of stem activities on 6th grade students’ conceptual development of sound. *Journal of Baltic Science Education, 20*(1), 21–37. 10.33225/jbse/21.20.21

DeJarnette, N. (2018). Implementing STEAM in the early childhood classroom. *European Journal of STEM Education, 3*(3), 18. 10.20897/ejsteme/3878

Dejonckheere, P., de Wit, N., van de Keere, K., & Vervaet, S. (2016). Exploring the classroom: Teaching science in early childhood. *European Journal of Educational Research, 5*(3), 149–164. 10.12973/eu-jer.5.3.149

Dilek, H., Tasdemir, A., Konca, A., & Baltaci, S. (2020). Preschool children’s science motivation and process skills during inquiry-based STEM activities*. Journal of Education in Science, Environment and Health, 6*(2), 92–104. 10.21891/jeseh.673901

Ergün, A., & Külekci, E. (2019). The effect of problem based STEM education on the perception of 5th grade students of engineering, engineers and technology. *Pedagogical Research, 4*(3), 1-15.

Firdaus, A. R., Wardani, D. S., Altaftazani, D. H., Kelana, J. B., & Rahayu, G. D. S. (2020). Mathematics learning in elementary school through engineering design process method with STEM approach. *Journal of Physics: Conference Series, 1657*(1). 10.1088/1742-6596/1657/1/012044

Fleer, M. (2021). Re-imagining play spaces in early childhood education: Supporting girls’ motive orientation to STEM in times of COVID-19. *Journal of Early Childhood Research, 19*(1), 3–20. 10.1177/1476718X20969848

Fridberg, M., & Redfors, A. (2021). Teachers’ and children’s use of words during early childhood STEM teaching supported by robotics. *International Journal of Early Years Education*. 10.1080/09669760.2021.1892599

Gold, Z., Elicker, J., Kellerman, A., Christ, S., Mishra, A., & Howe, N. (2021). Engineering play, mathematics, and spatial skills in children with and without disabilities. *Early Education and Development, 32*(1), 49–65. 10.1080/10409289.2019.1709382

Graves, L., Hughes, H., & Balgopal, M. (2016). Teaching STEM through horticulture: Implementing an edible plant curriculum at a STEM-centric elementary school. *Journal of Agricultural Education, 57*(3), 192–207.

Greca Dufranc, I. M., García Terceño, E. M., Fridberg, M., Cronquist, B., & Redfors, A. (2020). Robotics and early-years STEM education: The botSTEM framework and activities. *European Journal of STEM Education, 5*(1), 1–13. 10.20897/ejsteme/7948

He, X., Li, T., Turel, O., Kuang, Y., Zhao, H., & He, Q. (2021). The impact of STEM education on mathematical development in children aged 5-6 years. *International Journal of Educational Research, 109*, 101795. 10.1016/j.ijer.2021.101795

Hong, J.-C., Ye, J.-H., Ho, Y.-J., & Ho, H.-Y. (2020). Developing an inquiry and hands-on teaching model to guide STEAM lesson planning for kindergarten children. *Journal of Baltic Science Education, 19*(6), 908–922.

Hudson, P., English, L., Dawes, L., King, D., & Baker, S. (2015). Exploring links between pedagogical knowledge practices and student outcomes in STEM education for primary schools. *Australian Journal of Teacher Education, 40*(6), 1–19.

John, M., Sibuma, B., Wunnava, S., Anggoro, F., & Dubosarsky, M. (2018). An iterative participatory approach to developing an early childhood problem-based STEM curriculum. *European Journal of STEM Education, 3*(3), 1–12.

Koul, R., Fraser, B., & Nastiti, H. (2018). Transdisciplinary instruction: Implementing and evaluating a primary-school STEM teaching model. *International Journal of Innovation in Science and Mathematics Education, 26*(8), n/a.

Kuo-Ting, H., Ball, C., Cotten, S., & LaToya, O. (2020). Effective experiences: A social cognitive analysis of young students’ technology self-efficacy and STEM attitudes. *Social Inclusion, 8*(2), 213–221. 10.17645/si.v8i2.2612

Laksmiwati, P. A., Padmi, R. S., & Salmah, U. (2020, July). Elementary school teachers’ perceptions of STEM: What do teachers perceive? *Journal of Physics: Conference Series, 1581*(1).

Le Thi Xinh; Bui Van Hong (2021). STEM teaching skills of primary school teachers: The current situation in Ho Chi Minh City, Vietnam. *Journal of Education and e-Learning Research, 8*(2): 149–157.

Lin, X., Yang, W., Wu, L., Zhu, L., Wu, D., & Li, H. (2021). Using an inquiry-based science and engineering program to promote science knowledge, problem-solving skills and approaches to learning in preschool children. *Early Education and Development, 32*(5), 695–713. 10.1080/10409289.2020.1795333

Malcok, B., & Ceylan, R. (2021). The effects of STEM activities on the problem-solving skills of 6-year-old preschool children. *European Early Childhood Education Research Journal*. 10.1080/1350293X.2021.1965639

Malone, K., Tiarani, V., Irving, K., Kajfez, R., Lin, H., Giasi, T., & Edmiston, B. (2018). Engineering Design Challenges in Early Childhood Education: *Effects on Student Cognition and Interest, 3*(3).

Master, A., Cheryan, S., & Meltzoff, A. N. (2017). Social group membership increases STEM engagement among preschoolers. *Developmental Psychology, 53*(2), 201–209. 10.1037/dev0000195

Miller, J. (2019). STEM education in the primary years to support mathematical thinking: Using coding to identify mathematical structures and patterns*. ZDM Mathematics Education, 51*, 915–927. 10.1007/s11858-019-01096-y

Ndijuye, L., & Pambas, B. (2020). STEM starts early: Views and beliefs of early childhood education stakeholders in Tanzania. *Journal of Childhood, Education & Society, 1*(1), 29–42. 10.37291/2717638X.20201128

Onal, N., & Kirmizigul, A. (2022). A Makey-Makey based STEM activity for children. *Science Activities,* *58*(4), 166–182.

Orak, S., Çilek, A., & Yilmaz, F. (2020). Adaptation of traditional children’s games to social studies course: STEM course design for teachers. *Cypriot Journal of Educational Sciences, 15*(6), 1422–1438. 10.18844/cjes.v15i6.4318

Parlakay, E. S., & Koç, Y. (2020). An investigation the effect of STEM practices on fifth grade students’ academic achievement and motivations at the unit “Exploring and Knowing the World of Living Creatures”. *International Journal of Progressive Education, 16*(1), 125–137.

Pila, S., Piper, A., Lauricella, A., & Wartella, E. (2020). Preschoolers’ STEM learning on a haptic enabled tablet. *Multimodal Technologies and Interaction, 4*(4), 87. 10.3390/mti4040087

Qiao, X. & Zhou, X. (2020). Research on the integration of STEM education into the rural elementary school science curriculum: An example from rural elementary schools in Western China. *Best Evidence in Chinese Education, 5*(1), 581–590. https://doi.org/10.2139/ssrn.3607636

Rochman, C., Nasudin, D., & Rokayah, R. (2019, October). Science literacy on science technology engineering and math (STEM) learning in elementary schools. *Journal of Physics: Conference Series, 1318*(1).

Rokayah, R., & Rochman, C. (2019, October). Challenges in science technology engineering and math (STEM) learning in elementary schools based on literacy of social science. *Journal of Physics: Conference Series, 1318*(1).

Saddhono, K., Sueca, I. N., Sentana, G. D. D., Santosa, W. H., & Rachman, R. S. (2020, July). The application of STEAM (science, technology, engineering, arts, and mathematics)-based learning in elementary school Surakarta district. *Journal of Physics: Conference Series, 1573*(1).

Sangngam, S. (2021). The development of early childhood students’ creative thinking problem solving abilities through STEM Education learning activities. *Journal of Physics: Conference Series, 1835*(1).

Sariçam, U., & Yildirim, M. (2021). The effects of digital game-based STEM activities on students’ interests in STEM fields and scientific creativity: Minecraft case. *International Journal of Technology in Education and Science, 5*(2), 166–192.

Schroeder, E., & Kirkorian, H. (2016). When seeing is better than doing: Preschoolers’ transfer of STEM skills using touchscreen games. *Frontiers in Psychology, 7*(1377). 10.3389/fpsyg.2016.01377

Seage, S., & Türegün, M. (2020). The effects of blended learning on STEM achievement of elementary school students. *International Journal of Research in Education and Science, 6*(1), 133–140.

Shimwell, J., DeWitt, J., Davenport, C., Padwick, A., Sanderson, J., & Strachan, R. (2021). Scientist of the week: Evaluating effects of a teacher-led STEM intervention to reduce stereotypical views of scientists in young children. *Research in Science & Technological Education*. 10.1080/02635143.2021.1941840

Stephenson, T., Fleer, M., Fragkiadaki, G., & Rai, P. (2021). Teaching STEM through play: Conditions created by the conceptual playWorld model for early childhood teachers. *Early Years*. 10.1080/09575146.2021.2019198

Tippett, C., & Milford, T. M. (2017). Findings from a pre-kindergarten classroom: Making the case for STEM in early childhood education. *International Journal of Science and Mathematics Education, 15*(1), 67–86. 10.1007/s10763-017-9812-8

Türk, A., & Akcanca, N. (2021). Implementation of STEM in preschool education. *Journal of Educational Leadership and Policy Studies*.

Ugur-Erdogmus, F. (2021). How do elementary childhood education teachers perceive robotic education in kindergarten? A qualitative study. *Participatory Educational Research, 8*(2), 421–434. 10.17275/per.21.47.8.2

Ültay, N., Zivali, A., Yilmaz, H., Bak, H., Yilmaz, K., Topatan, M., & Kara, P. (2020). STEM-focused activities to support student learning in primary school science. *Journal of Science Learning, 3*(3), 156–164.

Üret, A., & Ceylan, R. (2021). Exploring the effectiveness of STEM education on the creativity of 5-year-old kindergarten children. *European Early Childhood Education Research Journal, 29*, 842–855. 10.1080/1350293X.2021.1913204

Wong, K., & Maat, S. M. (2020). The attitude of primary school teachers towards STEM education. *STEM Journal, 9*(3), 1243-1251.

Yavuz, Ü., & Yildiz Duban, N. (2021). Primary school students’ interests on professions and opinions on STEM implementations. *International Technology and Education Journal, 5*(1), 21–31.

Zhou, S. N., Zeng, H., Xu, S. R., Chen, L. C., & Xiao, H. (2019). Exploring changes in primary students’ attitudes towards science, technology, engineering and mathematics (Stem) across genders and grade levels. *Journal of Baltic Science Education, 18*(3), 466–480. 10.33225/jbse/19.18.466
